# Comments on Lu *et al.* Association between Self-Reported Global Sleep Status and Prevalence of Hypertension in Chinese Adults: Data from Kailuan Community. *Int. J. Environ. Res. Public Health* 2015, *12*, 488–503

**DOI:** 10.3390/ijerph120302901

**Published:** 2015-03-05

**Authors:** Yuee Huang, Phanuwat Sriyotha, Gholam Ali, Wenjie Sun

**Affiliations:** 1 Department of Preventive Medicine, School of Public Health, Wannan Medical University, Wuhu 241001, China; E-Mail: huangyewindow@163.com; 2 Laboratory for Environment and Health, School of Earth and Environment, Anhui University of Science and Technology, Huainan 231001, China; 3 Department of Global Environmental Health Sciences, School of Public Health and Tropical Medicine, Tulane University, 1440 Canal Street, New Orleans, LA 70112, USA; E-Mail: psriyoth@tulane.edu; 4 School of Medicine, Tulane University, 1430 Tulane Ave, New Orleans, LA 70112, USA; E-Mail: gali1@tulane.edu; 5 School of Food Science, Guangdong Pharmaceutical University, Zhongshan 528458, China; 6 School of Public Health and Tropical Medicine, Tulane University, 1440 Canal Street, New Orleans, LA 70112, USA

Lu *et al.* [1] examined the association between sleep status and prevalence of hypertension among Chinese adults varied by age and sex, using a cross-sectional study, including 5461 Chinese (4076 of them were male) aged 18 years or above, in Kailuan communities. Lu *et al.* claimed that short sleep duration was associated with hypertension only among Chinese men, and was attenuated after adjustment of sleep quality. However, the current conclusion of the study remains unclear.

Of note, four sub-communities were randomly selected from the Kailuan. Subjects aged 18 years or over among those four sub-communities were invited to participate in this study. According to the methods and a previous study [2], it is more likely this is an occupation-based study rather than a community-based study. This could partially explain why the sex ratio bias (male/female = 4076/1385) was significant in this study. Further, it could be questionable drawing such a conclusion with this potential bias. 

Previous studies showed that socioeconomic status (SES), which plays a key role in such a study [3], could affect sleep status, including duration, quality [4] and hypertension [5]. For example, a lower SES is strongly associated with a higher risk of hypertension [6,7]. The sleep status could therefore be affected by each effect or a combination of both. 

The study by Lu *et al.* underscores the association between sleep status and hypertension among the Chinese, without fully addressing the nature of the association. The conclusions would be more convincing if these matters were further quantified. 
